# Characterization and engineering of a DNA polymerase reveals a single amino-acid substitution in the fingers subdomain to increase strand-displacement activity of A-family prokaryotic DNA polymerases

**DOI:** 10.1186/s12860-019-0216-1

**Published:** 2019-08-09

**Authors:** Yvonne Piotrowski, Man Kumari Gurung, Atle Noralf Larsen

**Affiliations:** Department of Chemistry, Faculty of Science and Technology, SIVA Innovation Centre, Sykehusvegen 23, UiT - The Arctic University of Norway, 9037 Tromsø, Norway

**Keywords:** DNA polymerase, Enzyme engineering, Strand displacement, Molecular evolution, Isothermal amplification, Point-of-care

## Abstract

**Background:**

The discovery of thermostable DNA polymerases such as Taq DNA polymerase revolutionized amplification of DNA by polymerase chain reaction methods that rely on thermal cycling for strand separation. These methods are widely used in the laboratory for medical research, clinical diagnostics, criminal forensics and general molecular biology research. Today there is a growing demand for on-site molecular diagnostics; so-called ‘Point-of-Care tests’. Isothermal nucleic acid amplification techniques do not require a thermal cycler making these techniques more suitable for performing Point-of-Care tests at ambient temperatures compared to traditional polymerase chain reaction methods. Strand-displacement activity is essential for such isothermal nucleic acid amplification; however, the selection of DNA polymerases with inherent strand-displacement activity that are capable of performing DNA synthesis at ambient temperatures is currently limited.

**Results:**

We have characterized the large fragment of a DNA polymerase I originating from the marine psychrophilic bacterium *Psychrobacillus* sp*.* The enzyme showed optimal polymerase activity at pH 8–9 and 25–110 mM NaCl/KCl. The polymerase was capable of performing polymerase as well as robust strand-displacement DNA synthesis at ambient temperatures (25–37 °C). Through molecular evolution and screening of thousand variants we have identified a single amino-acid exchange of Asp to Ala at position 422 which induced a 2.5-fold increase in strand-displacement activity of the enzyme.

Transferring the mutation of the conserved Asp residue to corresponding thermophilic homologues from *Ureibacillus thermosphaericus* and *Geobacillus stearothermophilus* also resulted in a significant increase in the strand-displacement activity of the enzymes.

**Conclusions:**

Substituting Asp with Ala at positon 422 resulted in a significant increase in strand-displacement activity of three prokaryotic A-family DNA polymerases adapted to different environmental temperatures i.e. being psychrophilic and thermophilic of origin. This strongly indicates an important role for the 422 position and the O1-helix for strand-displacement activity of DNA polymerase I. The D422A variants generated here may be highly useful for isothermal nucleic acid amplification at a wide temperature scale.

**Electronic supplementary material:**

The online version of this article (10.1186/s12860-019-0216-1) contains supplementary material, which is available to authorized users.

## Background

DNA polymerases have been classified into seven families (A, B, C, D, X, Y, RT) based on their amino-acid sequence and structural homology [1]. These different families have distinct structural and functional properties needed to fulfill their different biological roles in nucleic-acid metabolism. The A-family DNA polymerases include both, replicative and repair polymerases. Prokaryotic A-family DNA polymerases, referred to as polymerase I, have two functional domains encoded within the same polypeptide chain, a 5′-3′ polymerase domain and a 5′-3′ exonuclease domain unique among all DNA polymerases (reviewed in [2]). In addition, some polymerase I enzymes also contain a proofreading 3′-5′ exonuclease domain, the main function of which is to remove errors during DNA replication, e.g. *Escherichia coli* DNA polymerase I (*E. coli*, reviewed in [2]).

Various A-family DNA polymerases are extensively used for in vitro amplification of DNA in molecular biology and diagnostic applications [3, 4], exemplified by the Taq DNA polymerase which is famous as the enzyme originally used in polymerase chain reaction (PCR, [5]). Other well-characterized enzymes from this family include the large fragment (LF) of *E. coli* DNA polymerase I, also known as the Klenow fragment [6], and the LF of *Geobacillus stearothermophilus* polymerase I (Gbst pol I LF, [7]). Gbst pol I LF is also able to perform strand displacement (SD) where the complement strand downstream of the polymerization direction is displaced simultaneously with nucleotide addition.

The structure of a DNA polymerase I, can be described in terms of a human right hand, with three subdomains referred to as the thumb, fingers, and palm (reviewed in [2]). Kaushik et al. [8] showed in their study that residues in the O- and O1-helix of the fingers subdomain are important for the polymerase function. A later study by Singh et al. [9] indicated that residues particular present in the O1-helix are essential for strand-displacement synthesis. The property of strand displacement allows Gbst pol I LF to be used in various isothermal nucleic acid amplification techniques (INAATs) such as loop-mediated isothermal amplification (LAMP, [10]) as strand separation is induced by the enzyme itself, rather than heat denaturation as used in PCR.

Globally, there is a high demand to monitor and diagnose critical infectious diseases. Continuous development of on-site molecular diagnostic tests, recently referred to as Point-of-Care (POC) tests, are needed to rapidly identify a specific pathogen and provide information on susceptibility to antimicrobial agents directing appropriate treatment [11]. The characteristics of an ideal new POC diagnostic test, valid also for low-resource settings, should meet the ASSURED criteria. The acronym ASSURED was originally coined at a 2003 WHO Special Programme for Research and Training in Tropical Diseases (WHO/TDR, [12]).

PCR meets necessary diagnostic requirements in terms of specificity, sensitivity and rapidity, but involves several steps and requires trained skilled technical personnel to perform sample preparation, DNA amplification and detection. In addition, PCR needs an accurate thermal cycler to perform the PCR reactions. In a POC setting, INAAT represents an enabling technology with the potential to offer rapid, sensitive and specific molecular diagnosis of infectious diseases aiming at meeting the ASSURED criteria (reviewed in [13]). In many of these methods, efficient target amplification relies on the inherent SD activity of the DNA polymerase used in the amplification step [14, 15]. Most of the currently used A-family DNA polymerases on the market, e.g. from *Bacillus stearothermophilus* and *Bacillus smithii*, have optimal performance at 60–65 °C and are less efficient in isothermal nucleic acid amplification at ambient temperatures required in POC settings.

In the present study we have recombinantly produced and characterized the large fragment of *Psychrobacillus* sp. DNA polymerase I (PB pol I LF), demonstrating that this enzyme exhibits efficient polymerase and SD activity at ambient temperatures. We have further improved this native SD activity through molecular evolution by introducing a single-point mutation in the fingers subdomain resulting in an increase of 150% (2.5 fold). Altering the equivalent residue in two thermostable A-family DNA polymerases resulted in a significant increase also in their SD activity (2.1 to 2.4 fold). We believe this study will contribute to the understanding of strand-displacement DNA synthesis by A-family DNA polymerases, potentially spurring development of new POC tests based on polymerase-driven isothermal amplification techniques.

## Results and discussion

**Fig. 1:**
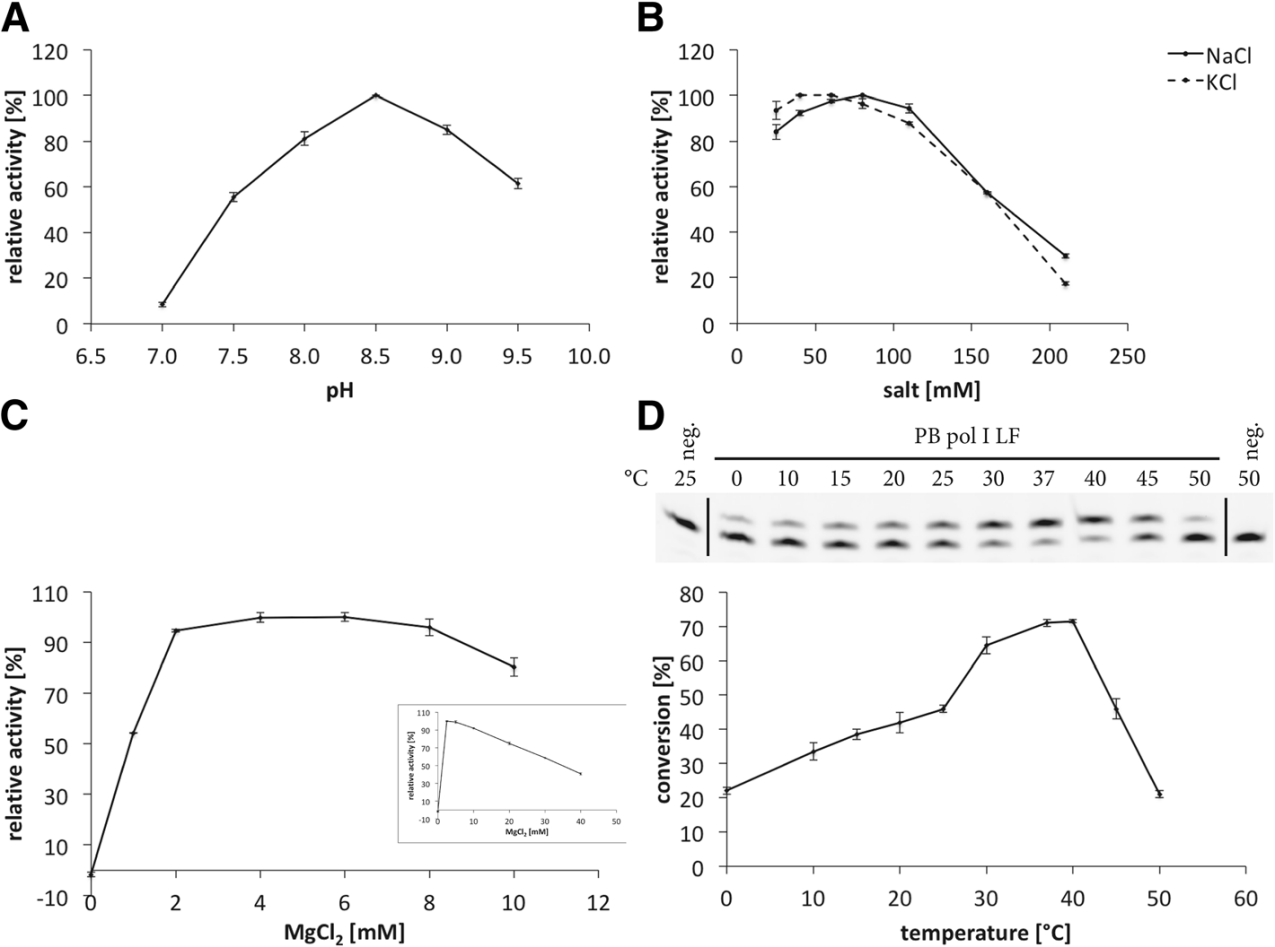
A-D Basic characterization of polymerase I large fragment from *Psychrobacillus* sp. The effect of pH (**a**), salt (**b**) and Mg^2+^ (**c**, inset showing polymerase activity at 0–40 mM MgCl_2_) have been measured with the time-resolved molecular beacon assay at 25 °C. The increase in Fluorescein fluorescence, i.e. enzyme activity, has been measured as relative fluorescence units (RFUs). For the reactions 37.5 ng protein were used. Effect of temperature (**d**) on the polymerase activity was tested with the single-nucleotide incorporation endpoint assay and densitometric analysis of the bands after denaturing polyacrylamide gel electrophoresis (12% polyacrylamide/7 M urea). For the reactions 180 pg protein were used. The gel is shown on top (neg. = negative control) and the evaluation of the densitometry at the bottom

**Fig. 2:**
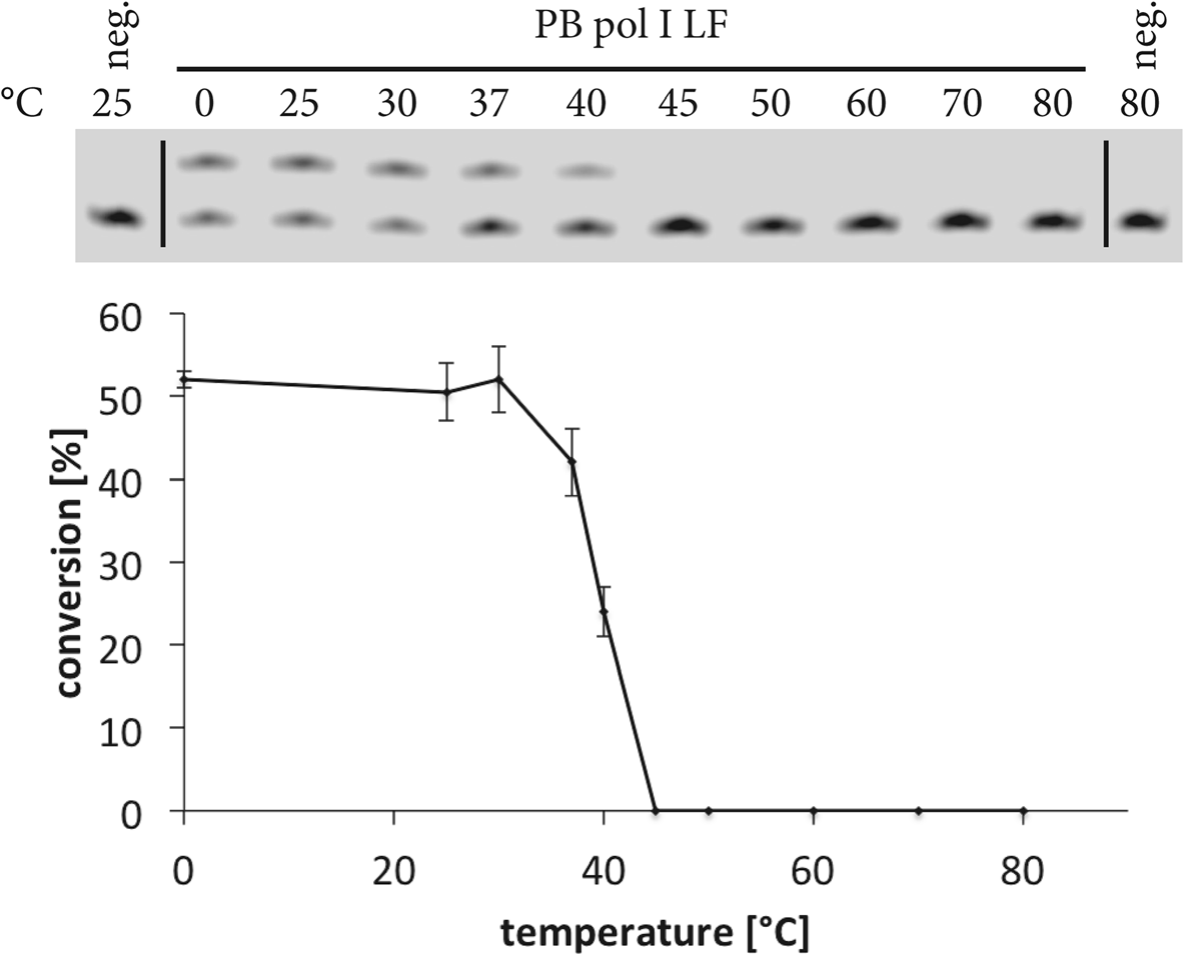
Temperature stability of polymerase I large fragment from *Psychrobacillus sp.* The thermal stability has been measured using the single-nucleotide incorporation endpoint assay and densitometric analysis (line chart at the bottom) of the bands after denaturing polyacrylamide gel electrophoresis (12% polyacrylamide/7 M urea, gel on top, neg. = negative control). After incubation of the reaction set-ups at the respective temperature the enzymatic reaction has been performed in 50 mM BIS-Tris propane pH 8.5 (at 25 °C), 100 mM NaCl, 5 mM MgCl_2_, 1 mM DTT, 0.2 mg/ml BSA and 2% glycerol. For the reactions 180 pg protein were used

The effect of pH, salt (NaCl, KCl) and Mg^2+^ on the polymerase activity were determined for PB pol I LF using a time-resolved polymerase activity assay with a molecular beacon substrate (see Methods). PB pol I LF showed highest polymerase activity at pH 8.5 and in the presence of 40–110 mM NaCl or 25–80 mM KCl (Fig. 1a and b). PB pol I LF had an absolute requirement for Mg^2+^ to perform DNA synthesis, hence no polymerase activity could be detected in the absence of Mg^2+^, and showed a decrease in polymerase activity at Mg^2+^ concentrations > 8 mM (Fig. 1c inset). PB pol I LF exhibited optimal performance at [Mg^2+^] in the range of 2–8 mM (Fig. 1c). The temperature optimum for polymerase activity and effect of temperature on the stability of PB pol I LF were determined using the single-nucleotide incorporation endpoint assay (see Methods). PB pol I LF showed moderate activity between 25 ° and 40 °C, with an optimum at 37 °C (Fig. 1d). PB pol I LF was rapidly inactivated when incubated at temperatures above 40 °C (Fig. 2).

**Fig. 3:**
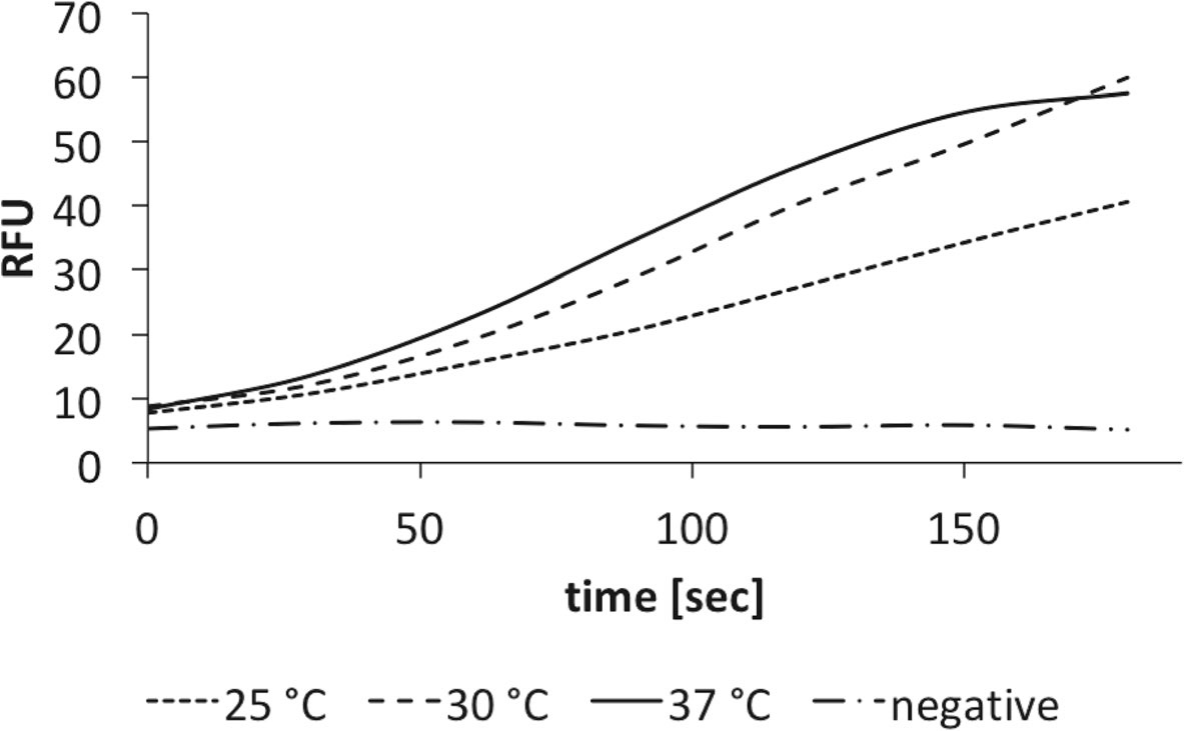
Strand-displacement activity of polymerase I large fragment from *Psychrobacillus* sp. at various temperatures. The strand-displacement activity assays have been performed in 50 mM BIS-Tris propane pH 8.5, 100 mM NaCl, 5 mM MgCl_2_, 1 mM DTT, 0.2 mg/ml BSA and 2% glycerol. The increase in TAMRA fluorescence, i.e. enzyme activity, has been measured as relative fluorescence units (RFUs). For the reactions 100 ng protein were used. “Negative” represents the sample where no enzyme has been added and thus the negative control, performed at 25 °C

**Fig. 4:**
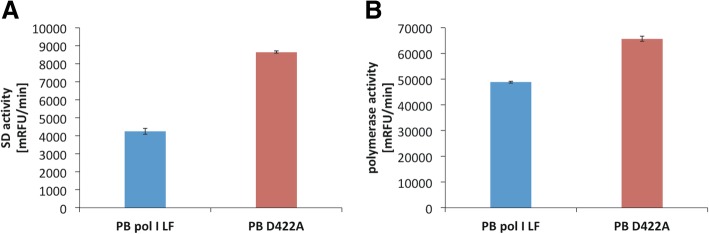
SD (**a**) and polymerase (**b**) activity analysis of polymerase I large fragment from *Psychrobacillus* sp. (PB pol I LF) and its D422A variant (PB D422A). Activities have been measured using the time-resolved strand-displacement and polymerase activity assay, respectively, at 25 °C in 50 mM BIS-Tris propane pH 8.5, 100 mM NaCl, 5 mM MgCl_2_, 1 mM DTT, 0.2 mg/ml BSA and 2% glycerol. The increase in TAMRA and Fluorescein fluorescence, i.e. SD and polymerase activity, respectively, has been measured as relative fluorescence units over time and is depicted as thousandth (milli) relative fluorescence unit per minute (mRFU/min). For SD 63 ng of each protein and for polymerase activity 36 ng of each protein were used

One of the most important properties for a polymerase-driven isothermal nucleic acid amplification method is the ability of the polymerase to perform strand displacement of dsDNA. Using the SD activity assay as described in Methods, PB pol I LF was demonstrated to perform strand-displacement DNA synthesis at 25 °C, 30 °C and 37 °C (Fig. 3). In order to gain information about residues involved in SD DNA synthesis and to develop a more potent enzyme for use in isothermal amplification methods we sought to increase the SD activity of the enzyme by molecular evolution. A thousand variants from the generated evolution library (see Methods) were expressed recombinantly, semi-purified and screened for SD activity using the time-resolved strand-displacement activity assay. Fourteen variants showed a significant increase in SD activity (> 1.5 fold) compared to the wild type enzyme of PB pol I LF. These variants were produced in larger scale (500 ml) and purified to homogeneity (> 95% purity determined by SDS-PAGE, Additional file 1). Out of the fourteen variants three still showed a significant increase in SD activity. The focus of this article is on the variant that showed the highest increase in SD activity of 150% (2.5 fold) at 25 °C (Fig. 4a). Sequencing analysis revealed a single base exchange at position 1265 from adenine to cytosine. The exchange of this base in the second position of the triplet originally encoding for an aspartate residue (G*A*T) led to a substitution to an alanine residue (G*C*T) at position 422 in the protein sequence of PB pol I LF (Additional file 2). The effect of the D422A substitution on polymerase activity was less pronounced and showed only an increase of about 1.4 fold (Fig. 4b), indicating that the D422A substitution mostly affected the SD function of the enzyme. Several other amino-acid substitutions have been tested in position 422, i.e. Ser, Lys, Val, Leu, Asn. These mutations cover the effect of substituting the Asp residue to other (larger) hydrophobic residues and polar residues of different length as well as a positive charged residue. All mutations resulted in improvement in the strand-displacement activity (Additional file 3), indicating that substituting the negatively charged Asp residue is beneficial for the strand-displacement capacity of the enzyme. The D422A mutation provides the substitution with the best enzyme performance.

To investigate whether the increase in SD activity induced by the D422A substitution was specific for PB pol I LF only, or whether 422 is an important position for SD DNA synthesis in other A-family DNA polymerases, a search for homologous proteins using Protein BLAST [16] was performed. The large fragment of DNA polymerase I from *Ureibacillus thermosphaericus* (Ubts pol I LF) and *Geobacillus stearothermophilus* (Gbst pol I LF) were chosen as thermophilic representatives. Ubts pol I LF and Gbst pol I LF have sequence identities of 60 and 67% with PB pol I LF, respectively.

**Fig. 5:**
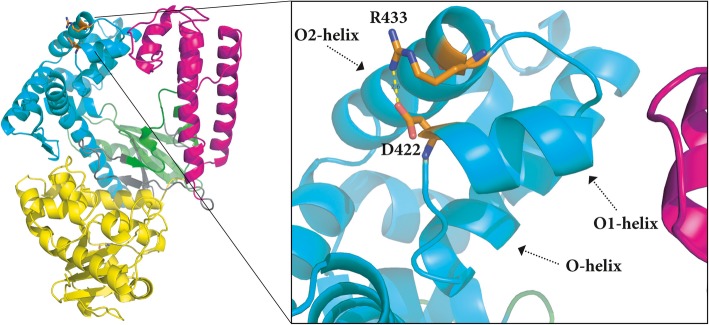
Cartoon representation of *Geobacillus stearothermophilus* polymerase I large fragment (PDB code 1XWL). Left showing the subdomains of the DNA polymerase large fragment: fingers (cyan), thumb (magenta) and palm (green). Right showing a close-up view of D422 residue (orange) located on the O1-helix and R433 residue (orange) located on the O2-helix and the distance measurement between them. The cartoon representation has been generated in PyMOL (The PyMol Molecular Graphics System, Version 2.0 Schrödinger, LLC)

**Fig. 6:**
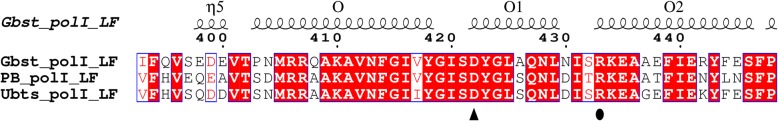
Section of sequence alignment between DNA polymerase I large fragment from *Geobacillus stearothermophilus* (Gbst_polI_LF), *Ureibacillus thermosphaericus* (Ubts_polI_LF) and *Psychrobacillus sp.* (PB_polI_LF) with secondary structure elements from Gbst pol I LF on top (PDB code 1XWL). The sequence alignment covers the O-helix, O1-helix and O2-helix including the conserved aspartate residue at position 422 (black triangle) and arginine residue at position 322 (black circle). The multiple sequence alignment has been generated with Clustal Omega [17] and illustrated including secondary structure information with ESPript 3.0 [18]

**Fig. 7:**
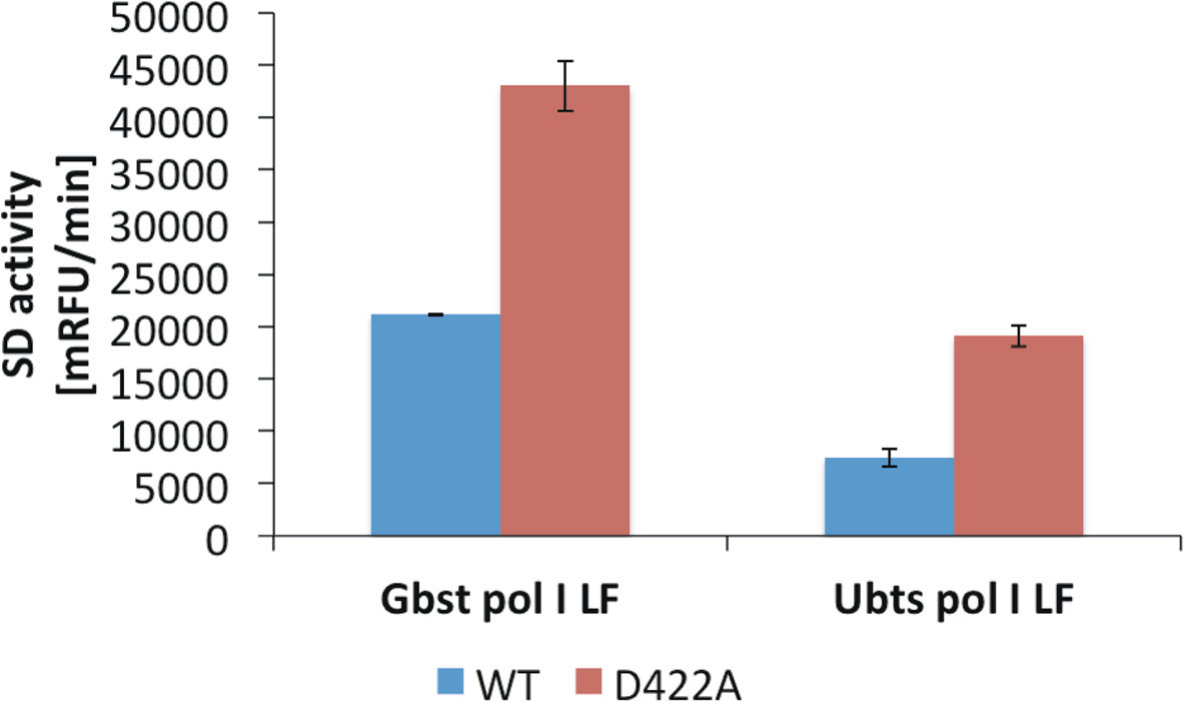
Strand-displacement activity of polymerase I large fragment from *Geobacillus stearothermophilus* (Gbst pol I LF) and *Ureibacillus thermosphaericus* (Ubts pol I LF) (wild type, WT, blue) and their respective D422A variants (red). Time resolved strand-displacement assays were performed at 37 °C in 20 mM Tris pH 7.9 (at 25 °C), 100 mM KCl, 10 mM (NH_4_)_2_SO_4_, 2 mM MgSO_4_, 0.1% Triton X-100. The increase in TAMRA fluorescence, i.e. enzyme activity, has been measured as relative fluorescence units (RFUs). Fifty nanogram Gbst pol I LF and 100 ng Ubts pol I LF were used in the reaction setup

Examination of the three-dimensional structure of Gbst pol I LF (PDB code: 1XWL) revealed that the amino-acid residue corresponding to the 422 position resides on a short α-helix, i.e. O1-helix. This α-helix is comprised of 44 amino-acid residues and is part of the fingers subdomain of the DNA polymerase I large fragment (Fig. 5, [7]) directed towards the thumb subdomain. According to the right-hand rule one can compare it to a line passing the tip of the index and middle finger. The neighboring secondary structure elements in three-dimensional space of O1-helix are the O- and O2-helix, all together forming a 3-helix bundle. Limiting the alignment of PB pol I LF, Gbst pol I LF and Ubts pol I LF to this 3-helix bundle (PB pol I LF aa403-aa446, Fig. 6) revealed high sequence identity among the selected sequences. The aspartate residue at position 422 of PB pol I LF is conserved in all three polymerases (black triangle in Fig. 6). We changed the respective aspartate residue to an alanine residue in Gbst pol I LF and Ubts pol I LF by site-directed mutagenesis. The respective D422A variants of Gbst pol I LF and Ubts pol I LF showed a 2.1 and 2.4 fold increase in SD activity, respectively, when compared to the wild type enzymes (Fig. 7).

**Fig. 8:**
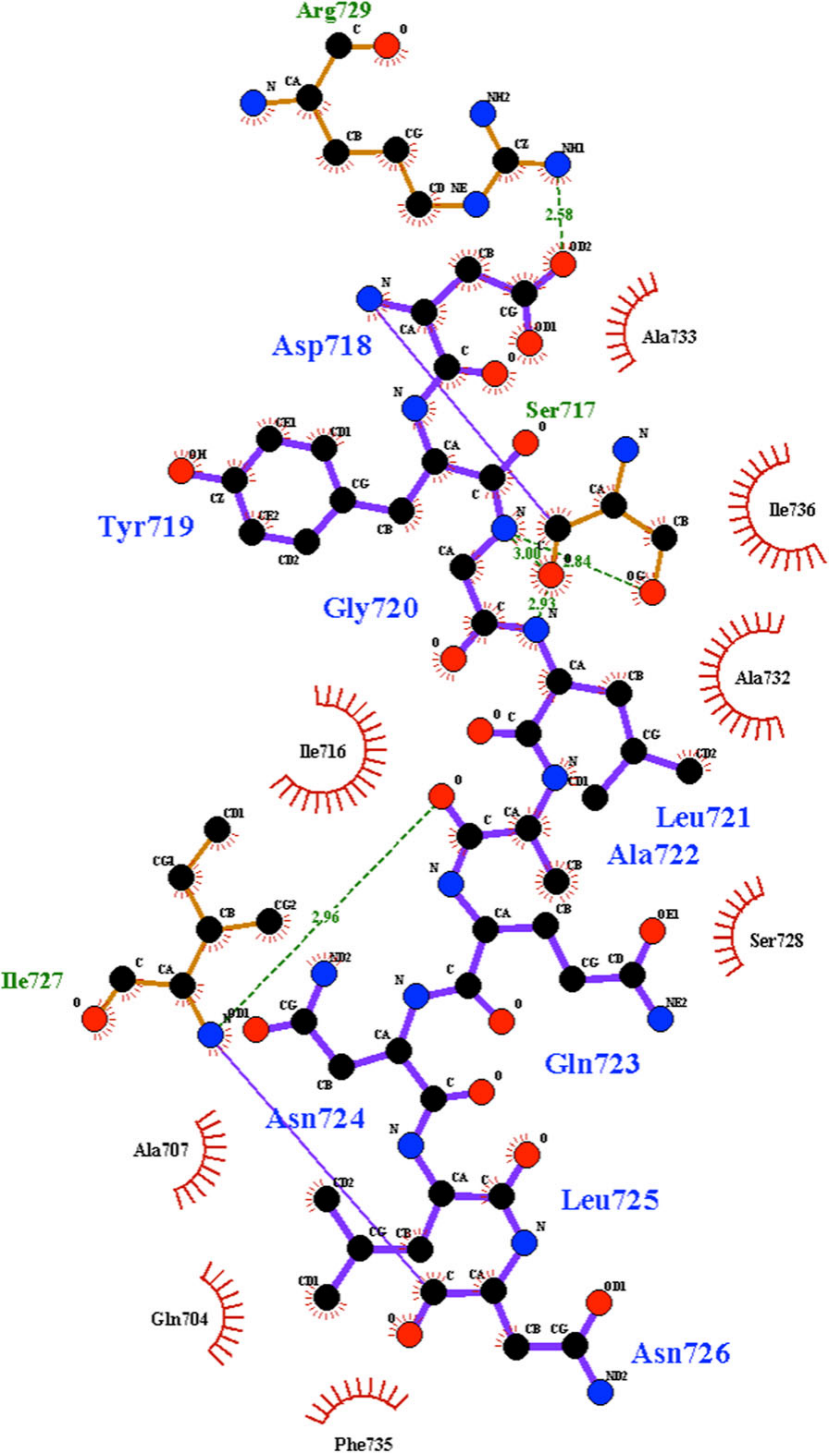
Schematic diagram generated with LIGPLOT [19] showing the interactions of amino-acid residues residing on O1-helix with neighboring secondary structure elements of Gbst pol I LF (PDB code 1XWL). Asp718 (numbering of the full-length protein of Gbst pol I) corresponds to Asp422 (numbering of PB pol I LF) and Arg729 corresponds to Arg433, respectively. Hydrogen bonds are indicated by dashed lines. Hydrophobic contacts are shown by an arc with spokes radiating towards the ligand atom of contact

The three-dimensional structure of Gbst pol I LF (PDB: 1WXL) shows that the interaction of the side chain of D422 with the side chain of R433 located on O2-helix, is the only direct interaction within the amino-acid sequence covered by the 3-helix bundle. The distance between NH1 of R433 and OD1 of D422 is 2.58 Å, indicating a salt bridge. Other than the salt bridge between D422 and R433, interactions within the 3-helix bundle are based on hydrogen bonds between the backbone atoms or hydrophobic interactions (Fig. 8). Substitution of the Asp side chain with a non-anionic side chain would disrupt a possible salt bridge forming with the Arg side chain in the 3-helix bundle motif rendering a more flexible region. Arg433 is conserved in the three polymerases of this study (black circle in Fig. 6), thus the observed increase in strand-displacement activity of the enzymes may be due to a local increase in flexibility.

In this study we have shown that changing the negatively charged aspartic acid to alanine at position 422 led to a significant increase in the ability of three A-family DNA polymerase large fragments to perform strand-displacement DNA-synthesis.

## Conclusion

The large fragment of DNA polymerase I from *Psychrobacillus sp.* has efficient polymerase and robust strand-displacement activity at low-moderate temperature and is thus a well-suited enzyme for DNA synthesis in isothermal amplification technologies at ambient temperatures. The D422A variant identified after molecular evolution of PB pol I LF possessed a 2.5 fold higher SD activity at 25 °C potentially improving polymerase driven INAAT at ambient temperatures. Our results further show that SD activity of the thermophilic Ubts and Gbst pol I LF could be increased as well by their respective D422A variants broadening the benefit of the discovered variant to INAAT methods also at higher temperatures such as LAMP.

## Methods

### Cloning of the gene encoding PB polymerase I large fragment

The gene encoding DNA polymerase I from *Psychrobacillus* sp. (Additional file 4) was cloned into the vector pET151/D-TOPO® using the Gateway® Technology (Thermo Fisher). The starting material for the polymerase chain reaction was the genomic DNA of *Psychrobacillus* sp., kindly provided by Marcin M. Pierechod. The bacterium has been collected from marine biota on a cruise of the research vessel Jan Mayen (Norway) around the Lofoten, an archipelago in Northern Norway. The genomic DNA has been isolated using the bead-beating method with the MP Biomedicals™ FastPrep-24™ Classic Instrument (Thermo Fisher Scientific). By use of the forward and reverse primer (Table 1) the gene has been truncated to the so-called large fragment of the DNA polymerase I (Additional file 5), i.e. omitting the 5′-3′ exonuclease domain of the protein.

**Table 1:** Forward (forw) and reverse (rev) primer sequences for cloning of wild type enzymes (wt) and for site-directed mutagenesis (substitution of Asp by Ala at position 422, D422A) of DNA polymerase I large fragments

Primer	Sequence (5′ to 3′ direction)
*Psychrobacillus* sp.	
PB_wt_forw	CACCACAGAAGTAGCATTCGAGATTGTT
PB_wt_rev	TTACTTCGTGTCATACCAAGATGAACC
*Geoacillus stearothermophilus*	
Gbst_wt_forw	ACCATCATGGATCCGGCGCCAAAATGGCATTTACCCT
Gbst_wt_rev	CATCCGCCAAAACAGCCTTATTTGGCATCATACCAGG
Gbst_D422A_forw	GTGTATGGCATCAGCGCTTATGGTCTGGCACAG
Gbst_D422A_rev	CTGTGCCAGACCATAAGCGCTGATGCCATACAC
*Ureibacillus thermosphaericus*	
Ureibac_wt_forw	ACCATCATGGATCCGGCGCAGCACTGAGCTTTAAAAT
Ureibac_wt_rev	CATCCGCCAAAACAGCCTTATTTGGCATCGTACCAGG
Ureibac_D422A_forw	CTATGGCATCAGCGCTTATGGTCTGAGCC
Ureibac_D422A_rev	GGCTCAGACCATAAGCGCTGATGCCATAG

### Evolution library creation

To generate an evolution library of PB pol I LF a fragment thereof covering amino-acid residue 174 to 580, i.e. omitting the first third of the protein, was submitted to codon optimization and molecular evolution experiments (Gene™ Controlled Randomization technology, Thermo Fisher Scientific) with a default of an average number of 3.5 amino-acid residue mutations per construct in the pET-11a vector. According to the manufacturer the amplified library was digested with NheI/BamHI and ligated into the pET-11a vector. Ligation reactions were transformed into *E. coli* strain DH5a and the transformation rate was determined by plating of dilution series. The total number of transformants was 1.53 × 10^5^ cfu. The evolution library was received as glycerol stock preparation, i.e. total cells from the transformation were resuspended in 50% glycerol at 1.55 × 10^10^ cells/ml.

### Small-scale protein production and semi-purification in 96-well plate format

The evolution library from Thermo Fisher Scientific was received as glycerol stock preparation. These glycerol stocks consisted of the cloned library in pET-11a vector in DH5α cells. Plasmid isolation has been performed in 96-well format with PureLink™ Pro Quick96 Plasmid Purification Kit (Thermo Fisher Scientific) from single colonies after striking out the glycerol stock onto LB/Amp plates and overnight cultivation in 1.5 ml Luria Bertani (LB)/ampicillin (100 μg/ml) thereof. Subsequently the isolated plasmids, each representing a single variant of PB pol I LF with one or more mutations, have been transformed into in-house produced chemically competent Rosetta 2 (DE3) cells in 48-well format for recombinant protein production. For the overnight culture 1.5 ml LB/ampicillin (100 μg/ml) were inoculated with 5–6 colonies of each variant. After incubation overnight at 37 °C and 220 rpm 250 μl were transferred into 3 ml fresh Terrific Broth (TB)/ampicillin (100 μg/ml) media. Cells grew at 37 °C until OD_600 nm_ reached 0.5–1.0. Gene expression was then induced by addition of 0.1 mM IPTG and carried out at 15 °C, 220 rpm for 6–8 h. Cells were harvested by centrifugation with a plate rotor at 500 x *g* for 10 min. Cell pellets were resuspended in 1 ml 50 mM HEPES pH 7.5 (at 25 °C), 500 mM NaCl, 10 mM imidazole, 5% glycerol, 0.25 mg/ml lysozyme. Cell disruption was performed by sonication with the VCX 750 from Sonics® (pulse 1.0/1.0, 1 min, amplitude 25%). Subsequent semi-purification of the proteins was performed in 96-well plate format with His MultiTrap™ HP (GE Healthcare) according to the instructor’s manual. Proteins were eluted in 50 μl 50 mM HEPES pH 7.5 (at 25 °C), 500 mM NaCl, 500 mM imidazole, 5% glycerol. Protein concentration was determined with the Bradford assay [20] in 96-well format using 10 μl of semi-purified protein. During the whole procedure the wild type enzyme has been used as a control.

### Cloning of genes encoding polymerase I large fragment from *Geobacillus stearothermophilus* and *Ureibacillus thermosphaericus*

The codon-optimized genes encoding polymerase I large fragment from *Geobacillus stearothermophilus* (Gbst pol I LF, NCBI protein database: 3TAN_A) and *Ureibacillus thermosphaericus* (Ubts pol I LF, NCBI protein database: WP_016837139) were purchased from the Invitrogen GeneArt Gene Synthesis service from Thermo Fisher Scientific. The genes were cloned into the vector pTrc99a (encoding an N-terminal His_6_-tag) by FastCloning after Li et al. [21]. The corresponding substitution from Asp to Ala at position 422 (PB pol I LF) was introduced using the QuikChange II Site-Directed Mutagenesis Kit (Agilent Technologies) and confirmed by sequencing analysis. Primer sequences for cloning and site-directed mutagenesis are listed in Table 1.

### Recombinant protein production and purification PB pol I LF and its D422A variant

Recombinant protein production of PB pol I LF and its D422A variant was performed in Rosetta 2 (DE3) cells (Novagen®). Cells grew in TB/ampicillin (100 μg/ml) media and gene expression was induced at OD_600 nm_ = 1.0 by addition of 0.1 mM IPTG. Protein production was carried out at 15 °C, 180 rpm for 6–8 h. For protein purification the pellet of a 1-l cultivation was resuspended in 50 mM HEPES pH 7.5 (at 25 °C), 500 mM NaCl, 10 mM imidazole, 5% glycerol, 0.15 mg/ml lysozyme, 1 protease inhibitor tablet (cOmplete™, Mini, EDTA-free Protease Inhibitor Cocktail, Roche) and incubated on ice for 30 min. If not stated otherwise all steps during the protein purification have been performed either on ice or cooled at 4 °C. Cell disruption was performed by French press (1.37 kbar) and subsequently by sonication with the VCX 750 from Sonics® (pulse 1.0/1.0, 5 min, amplitude 25%). In the first step the soluble part of the His_6_-tagged protein present after centrifugation (48,384 x *g*, 45 min, 4 °C) was purified by immobilized Ni^2+^-affinity chromatography. After a wash step with 50 mM HEPES pH 7.5 (at 25 °C), 500 mM NaCl, 50 mM imidazole, 5% glycerol the protein was eluted at an imidazole concentration of 250 mM and further transferred into 50 mM HEPES pH 7.5 (at 25 °C), 500 mM NaCl, 10 mM MgCl_2_, 5% glycerol by use of a desalting column. The second step was cleavage of the tag by the TEV protease performed overnight at 4 °C in 50 mM Tris pH 8.0 (at 25 °C), 0.5 mM EDTA and 1 mM DTT. To separate the protein from the His_6_-tag and the His_6_-tagged TEV protease a second Ni^2+^-affinity chromatography has been performed in the third step in 50 mM HEPES pH 7.5 (at 25 °C), 500 mM NaCl, 5% glycerol. The tag-free protein eluted in the flow through after applying the TEV-cleavage reaction onto the column. The His_6_-tag and the His_6_-tagged TEV protease have been eluted from the column with 50 mM HEPES pH 7.5 (at 25 °C), 500 mM NaCl, 500 mM imidazole, 5% glycerol. The final protein solution was concentrated and stored with 50% glycerol at − 20 °C for activity assays.

### Recombinant protein production and purification Gbst and Ubts pol I LF

Gbst and Ubts pol I LF and their D422A variants have been produced recombinant in Rosetta 2 (DE3) cells (Novagen®). Cultivation of cells has been performed in LB/ampicillin (100 μg/ml) media and incubation at 37 °C. After induction of gene expression at OD_600 nm_ = 0.5 by addition of 0.5 mM IPTG, protein production was carried out at 37 °C for 4 h. If not stated otherwise all steps during the subsequent protein purification have been performed either on ice or cooled at 4 °C. The pellet of a 0.5-l cultivation was resuspended in 50 mM Tris pH 8.0 (at 25 °C), 300 mM NaCl, 1 mM EDTA, 1 mM DTT, 10 mM imidazole, 0.15 mg/ml lysozyme, 1 protease inhibitor tablet (cOmplete™, Mini, EDTA-free Protease Inhibitor Cocktail, Roche), incubated on ice for 30 min and then subjected to sonication with the VCX 750 from Sonics® (pulse 1.0/1.0, 15 min, amplitude 25%) for cell disruption. The soluble part of the His_6_-tagged protein present after centrifugation (48,384 x *g*, 45 min, 4 °C) was purified by immobilized Ni^2+^-affinity chromatography. After a wash step with 50 mM Tris pH 8.0 (at 25 °C), 300 mM NaCl, 1 mM EDTA, 1 mM DTT, 10 mM imidazole the protein was eluted with gradually increasing the imidazole to 500 mM. Fractions containing the protein were collected, and buffer exchange was performed into 20 mM Tris pH 7.1 (at 25 °C), 100 mM KCl, 2 mM DTT, 0.2 mM EDTA and 0.2% Triton X-100 by desalting. After concentration the final protein solution was stored with 50% glycerol at − 20 °C for activity assays.

### Single-nucleotide incorporation assay

For determination of optimal temperature for polymerase activity 10 μl reactions contained 30 nM substrate, fluorophore-labeled primer annealed to template DNA (Table 2), and 10 μM dATP. For PB pol I LF the reaction further contained 5 mM MgCl_2_ in 50 mM Tris pH 8.5, 100 mM NaCl, 1 mM DTT, 0.2 mg/ml BSA and 2% glycerol. The pH of the reaction buffer at room temperature was adjusted to pH 8.5 at the respective incubation temperature. The reactions were initiated by addition of protein solution and incubated for 15 min at various temperatures (0 °C–50 °C). As negative control protein dilution buffer (10 mM HEPES pH 7.5 (at 25 °C), 1% glycerol) has been used instead of protein solution.

**Table 2:** Oligonucleotide sequences for enzymatic assay substrates. [FAM]: derivative of the fluorophore Fluorescein, attached to position 5 of the thymidine ring; Dabcyl: N-[4-(4-dimethylamino)phenylazo] benzoic acid, dark quencher (non-fluorescent chromophore) attached to position 5 of the thymidine ring; Flc: Fluorescein, fluorophore attached to position 5 of the thymidine ring; [BHQ2]: Black Hole Quencher 2, dark quencher (non-fluorescent chromophore) – attached to the 5′ end via a phosphodiester bond; [TAMRA]: Carboxytetramethylrhodamine, attached to the 3′ end via a phosphodiester linkage

Oligonucleotide	Sequence (5′ to 3′ direction)
Single-nucleotide incorporation assay	
Template	ATTGAGTGGAGATAGTATCGTAGGGTAGTATTGGTGGATA
Primer	[FAM] TATCCACCAATACTACCCT
Polymerase activity assay	
Template	*GGCCCGT*^*Dabcyl*^AGGAGGAAAGGACATCTTCTAGCA*T*^*Flc*^*ACGGGCC*GTCAAGTTCATGGCCAGTCAAGTCGTCAGAAATTTCGCACCAC
Primer	GTGGTGCGAAATTTCTGAC
Strand-displacement activity assay	
Template	[BHQ2]ATTGAGTGGACAAAGTATCGTAGGGTAGTATTGGTGGATA
Reporter strand	CGATACTTTGTCCACTCAAT [TAMRA]
“Cold” primer	TATCCACCAATACTACCCT

To examine thermal stability of PB pol I LF 10 μl reactions contained 50 mM BIS-TRIS propane at pH 8.5 (at 25 °C), 100 mM NaCl, 5 mM MgCl_2_, 1 mM DTT, 0.2 mg/ml BSA and 2% glycerol. PB pol I LF was added to the reaction buffer, incubated at various temperatures (0 °C – 80 °C) for 15 min and afterwards cooled down on ice for 5 min. As negative control protein dilution buffer (10 mM HEPES pH 7.5 (at 25 °C), 1% glycerol) has been used instead of protein solution. The single-nucleotide extension reaction was initiated by addition of 30 nM substrate (Table 2) and 10 μM dATP. The mixture was incubated at 25 °C for 15 min.

Reactions were stopped by addition of 2.5 μl denaturing gel loading buffer (95% formamide, 10 mM EDTA, 0.1% xylene cyanol) and incubation at 95 °C for 5 min. For denaturing polyacrylamide gel electrophoresis (12% polyacrylamide/7 M urea) a sample volume of 6 μl was loaded onto the gel. Gel electrophoresis was performed in 0.5x TBE buffer (44.5 mM Tris, 44.5 mM boric acid, 1 mM EDTA) at 50 W for 1 h 15 min and the gel subsequently scanned for FAM with the PharosFX Plus Imager (Bio-Rad).

Enzyme activity was determined by densitometric measurement of bands representing the extended primer (intensity 1) and the unextended primer (intensity 0). Analysis of quantitative data has been performed using standard deviation. The relative conversion rate was calculated as follows:

conversion [%] = intensity 1/(intensity 0 + intensity 1)*100.

### Polymerase activity assay

The polymerase activity assay is based on a molecular beacon probe (modified from [22]). Fifty microliter reactions consisted of 200 nM substrate, primer annealed to template DNA consisting of fluorophore and quencher (Table 2), and 200 μM dNTPs (equimolar amounts of dATP, dGTP, dCTP and dTTP). For PB pol I LF the reaction further contained 5 mM MgCl_2_ in 50 mM BIS-Tris propane at pH 8.5 (at 25 °C), 100 mM NaCl, 1 mM DTT, 0.2 mg/ml BSA and 2% glycerol. For Gbst and Ubts pol I LF the reaction further contained 20 mM Tris pH 7.9 (at 25 °C), 100 mM KCl, 10 mM (NH_4_)_2_SO_4_, 2 mM MgSO_4_, 0.1% Triton X-100.

The activity assay was carried out at 25 °C and 37 °C, respectively, in black 96-well fluorescence assay plates (Corning®). The reaction was initiated by addition of protein solution. The increase in Fluorescein fluorescence was measured as relative fluorescence units (RFUs) in appropriate time intervals by exciting at 485 nm and recording emission at 518 nm. Measurements were performed in a SpectraMax® Gemini Microplate Reader (Molecular Devices). Analysis of quantitative data has been performed using standard deviation.

### Strand-displacement activity assay

Fifty microliter reactions consisted of 200 nM substrate, “cold” primer and reporter strand annealed to template DNA (Table 2), and 200 μM dNTPs (equimolar amounts of dATP, dGTP, dCTP and dTTP). For PB pol I LF and screening of variants from the evolution library the reaction further contained 5 mM MgCl_2_ in 50 mM BIS-Tris propane at pH 8.5 (at 25 °C), 100 mM NaCl, 1 mM DTT, 0.2 mg/ml BSA and 2% glycerol. For Gbst and Ubts pol I LF the reaction further contained 20 mM Tris pH 7.9 (at 25 °C), 100 mM KCl, 10 mM (NH_4_)_2_SO_4_, 2 mM MgSO_4_, 0.1% Triton X-100.

The activity assay was carried out at 25 °C and 37 °C, respectively, in black 96-well fluorescence assay plates (Corning®). The reaction was initiated by addition of protein solution. The increase in TAMRA fluorescence was measured as RFUs in appropriate time intervals by exciting at 525 nm and recording emission at 598 nm. Measurements were performed in a SpectraMax® M2^e^ Microplate Reader (Molecular Devices). Analysis of quantitative data has been performed using standard deviation.

### Mutagenesis, protein production and semi-purification of PB pol I LF 422 variants

Amino-acid substitutions at position 422 of PB pol I LF have been introduced using the QuikChange II Site-Directed Mutagenesis Kit (Agilent Technologies) and confirmed by sequencing analysis. Starting material for the mutagenesis reaction was the gene encoding PB D422A in the vector pET-11a. Recombinant protein production has been performed in Rosetta 2 (DE3) cells (Novagen®) in 25 ml TB/ampicillin (100 μg/ml) media. At OD_600 nm_ = 1.0 gene expression was induced by addition of 0.1 mM IPTG. Incubation temperature was lowered from 37 °C to 15 °C and protein production was carried out at 180 rpm for 6–8 h. Semi-purification has been performed with PureProteome™ Nickel Magnetic Beads (Millipore). Cells have been lysed by sonication with VCX 750 from Sonics® (pulse 1.0/1.0, 1 min, amplitude 20%) in 1 ml lysis buffer (50 mM HEPES pH 7.5 (at 25 °C), 500 mM NaCl, 5% glycerol, 150 μg lysozyme) and processed further according to manufacturer’s instructions (washing buffer: 50 mM HEPES pH 7.5 (at 25 °C), 500 mM NaCl, 5% glycerol). Final elution of the proteins has been performed with 50 μl elution buffer (50 mM HEPES pH 7.5 (at 25 °C), 500 mM NaCl, 500 mM imidazole, 5% glycerol). Protein concentrations have been determined using the Bradford assay [20]. SD activity of PB pol I wild type (Asp) and its variants containing amino-acid substitutions at position 422 has been determined using the time-resolved strand-displacement activity assay.

## Additional files

Additional file 1: SDS-PAGE analysis of semi-purified PB pol I LF and selected variants. Lane 1: Mark12™ Unstained Standard (Thermo Fisher Scientific), lane 2: D442A variant, lane 3: variant 2, lane 4: variant 3, lane 5: PB pol I LF. PB pol I LF and its variants have been purified from a 500-ml cultivation pellet by immobilized Ni^2+^-affinity chromatography including cleavage of the His_6_-tag by TEV protease. For each sample 11 μg of semi-purified protein have been loaded onto the gel. (TIF 3875 kb)

Additional file 2: Sequencing analysis after directed evolution of PB pol I LF. The diagram was generated with SnapGene® software (from GSL Biotech) and shows the triplet coding for alanine (GCT, blue background) after base exchange by random mutagenesis of the wild type sequence coding for an aspartate (GAT) at the respective position. (DOC 461 kb)

Additional file 3: The effect of amino-acid substitution at position 422 of PB pol I LF (Asp) on SD activity. Activity has been measured (in duplicates) using the time-resolved strand-displacement activity assay at 25 °C in 50 mM BIS-Tris propane pH 8.5, 100 mM NaCl, 5 mM MgCl_2_, 1 mM DTT, 0.2 mg/ml BSA and 2% glycerol. The increase in TAMRA fluorescence has been measured as relative fluorescence units over time and is depicted as specific SD activity as thousandth (milli) relative fluorescence unit per minute per μg protein (mRFU/min/μg). (DOCX 56 kb)

Additional file 4: Nucleotide sequence encoding PB pol I LF. (DOC 25 kb)

Additional file 5: Amino-acid sequence of PB pol I LF. (DOCX 43 kb)

## Data Availability

All data generated or analyzed during this study are included in this published article or available from the corresponding author on reasonable request.

## Abbreviations

BSA: bovine serum albumin
dNTPs: deoxynucleotide triphosphates
DTT: Dithiothreitol
EDTA: ethylenediaminetetraacetic acid
IPTG: Isopropyl β-D-1-thiogalactopyranosid
OD_600 nm_: optical density at 600 nm
SDS-PAGE: Sodium dodecyl sulfate polyacrylamide gel electrophoresis
