# Supplementary figures and images for: Characterization and engineering of a DNA polymerase reveals a single amino-acid substitution in the fingers subdomain to increase strand-displacement activity of A-family prokaryotic DNA polymerases
Source: BMC Mol Cell Biol. 2019 Aug 9;20:31. doi: 10.1186/s12860-019-0216-1 (PMC6688381; doi:10.1186/s12860-019-0216-1)

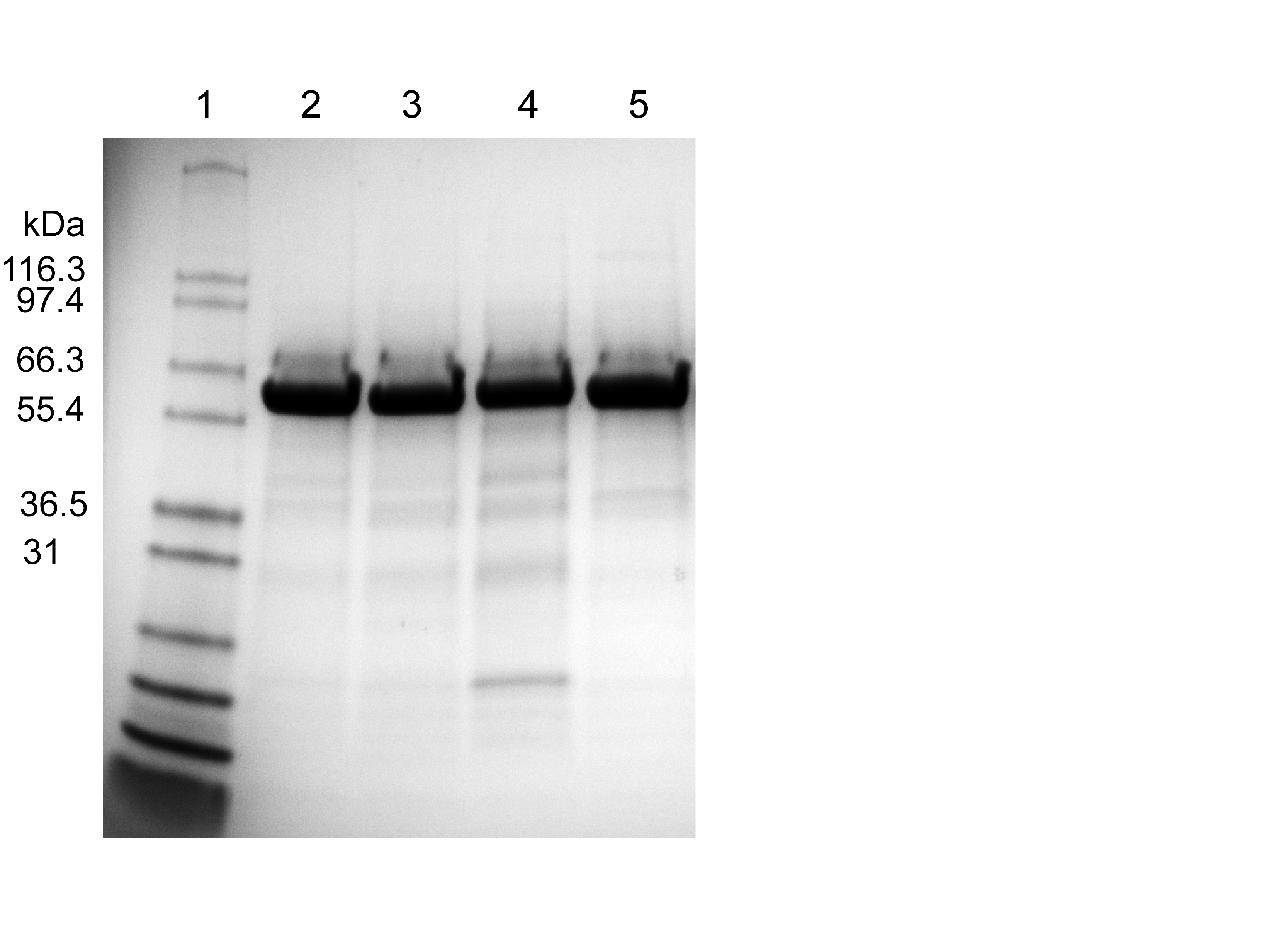

Supplement: Supplementary file 1 — SDS-PAGE analysis of semi-purified PB pol I LF and selected variants. Lane 1: Mark12™ Unstained Standard (Thermo Fisher Scientific), lane 2: D442A variant, lane 3: variant 2, lane 4: variant 3, lane 5: PB pol I LF. PB pol I LF and its variants have been purified from a 500-ml cultivation pellet by immobilized Ni2+-affinity chromatography including cleavage of the His6-tag by TEV protease. For each sample 11 μg of semi-purified protein have been loaded onto the gel. (TIF 3875 kb) [file 12860_2019_216_MOESM1_ESM.tif]

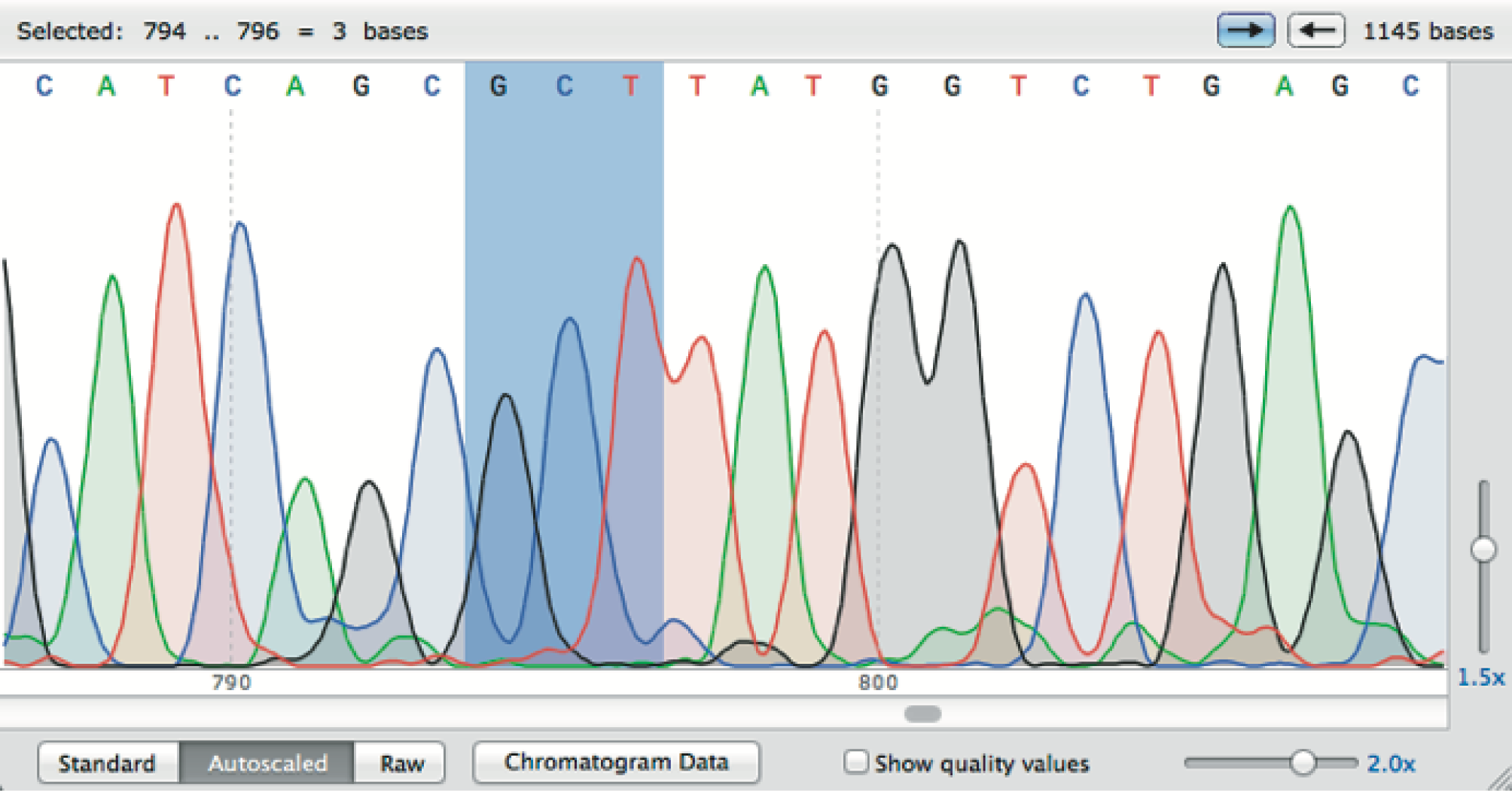

Supplement: Supplementary file 2 — Sequencing analysis after directed evolution of PB pol I LF. The diagram was generated with SnapGene® software (from GSL Biotech) and shows the triplet coding for alanine (GCT, blue background) after base exchange by random mutagenesis of the wild type sequence coding for an aspartate (GAT) at the respective position. (DOC 461 kb) [file 12860_2019_216_MOESM2_ESM.doc]

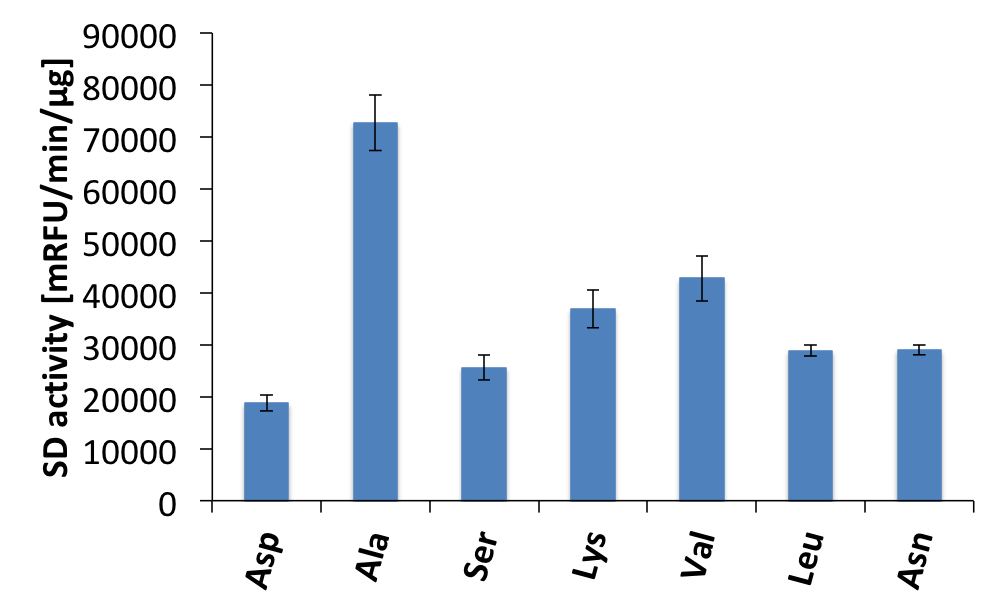

Supplement: Supplementary file 3 — The effect of amino-acid substitution at position 422 of PB pol I LF (Asp) on SD activity. Activity has been measured (in duplicates) using the time-resolved strand-displacement activity assay at 25 °C in 50 mM BIS-Tris propane pH 8.5, 100 mM NaCl, 5 mM MgCl2, 1 mM DTT, 0.2 mg/ml BSA and 2% glycerol. The increase in TAMRA fluorescence has been measured as relative fluorescence units over time and is depicted as specific SD activity as thousandth (milli) relative fluorescence unit per minute per μg protein (mRFU/min/μg). (DOCX 56 kb) [file 12860_2019_216_MOESM3_ESM.docx]
