# Supplementary material for: Characterization and engineering of a DNA polymerase reveals a single amino-acid substitution in the fingers subdomain to increase strand-displacement activity of A-family prokaryotic DNA polymerases
Source: BMC Mol Cell Biol. 2019 Aug 9;20:31. doi: 10.1186/s12860-019-0216-1 (PMC6688381; doi:10.1186/s12860-019-0216-1)
Supplement: Supplementary file 4 — Nucleotide sequence encoding PB pol I LF. (DOC 25 kb) [file 12860_2019_216_MOESM4_ESM.doc]

acagaagtagcattcgagattgttgaagaaattgactctacaatattagataaagtaatgtcagtccatttagaaatgtatgatgggcaatatcatacaagcgaattattaggtattgctttatcagatggagaaaagggttattttgctcctgctgatatagcttttcaatcgaaggatttttgttcttggttagaaaatgctacgaataaaaagtatttagcagactccaaagcaacacaagcagtgagtagaaaacataatgtgaatgtacatggagtggaattcgaccttcttttagcagcgtatatagtaaatcctgctatctcttcagaggatgttgctgctattgctaaagaatttggatattttaacttgctgacaaacgatagtgtttatgggaaaggtgccaaaaaaaccgcacctgaaatcgagaaaattgcagaacatgccgtaagaaaagcaagggctatttgggacttgaaagaaaagttagaagtaaaactggaagaaaatgaacaatatgcgttgtataaagaaatagagctaccgcttgcatctatccttggtacgatggaatcagatggggtgctggtggataaacaaattcttgtagaaatgggtcatgagcttaatattaagttacgagcgattgaacaagacatttatgcgttagctggtgaaacgtttaatattaattcacctaaacaattaggtgtaatactatttgaaaaaattggtcttacccctattaaaaagacaaaaacgggctattcaactgcagcagatgttttggaaaaactagcaagtgaacatgaaataatagagcaaattttactatatcgtcaattaggtaaactcaattccacatatatcgaaggattattaaaagagattcatgaagatgatgggaagatccatacccgatatcaacaagccctaacttcaactgggcgtttgagttcgatcaatccaaaccttcaaaatataccagttcgtttagaagaaggtagaaaaatacgtaaagcctttgttccttcacaaccgggatgggtaatgtttgcggcggattactctcaaattgaattgcgtgttcttgcccatatgtctgaggatgaaaacctggtagaagcttttaataatgatctggatattcatactaaaacggctatggatgtattccatgtggagcaggaagcagtaacgtccgatatgcgccgtgctgctaaggcagttaactttgggattgtgtatggtattagtgattatggtttatcacaaaacctagatattactagaaaagaagcggcgacatttatcgagaattatttaaatagcttcccaggtgtaaaaggatatatggatgatatcgttcaagatgcgaaacaaacaggctacgttacaacaattttgaatagacgaagatatttgcctgaaataacaagttctaactttaatctccgcagttttgcagaacgtactgctatgaatacaccaattcaagggagtgcagccgatattattaaaaaagcaatgatcgatatggcggaaagattaatatcagaaaatatgcagaccaaaatgctactacaagtacatgatgaattaatttttgaggctccaccagaggaaattgcaatgctagaaaaaatagtgccagaggtgatggaaaacgctattaaactgattgtacctttgaaagtggattatgcctttggttcatcttggtatgacacgaagtag
