# Supplementary material for: Characterization and engineering of a DNA polymerase reveals a single amino-acid substitution in the fingers subdomain to increase strand-displacement activity of A-family prokaryotic DNA polymerases
Source: BMC Mol Cell Biol. 2019 Aug 9;20:31. doi: 10.1186/s12860-019-0216-1 (PMC6688381; doi:10.1186/s12860-019-0216-1)
Supplement: Supplementary file 5 — Amino-acid sequence of PB pol I LF. (DOCX 43 kb) [file 12860_2019_216_MOESM5_ESM.docx]

TEVAFEIVEE IDSTILDKVM SVHLEMYDGQ YHTSELLGIA LSDGEKGYFA PADIAFQSKD FCSWLENATN KKYLADSKAT QAVSRKHNVN VHGVEFDLLL AAYIVNPAIS SEDVAAIAKE FGYFNLLTND SVYGKGAKKT APEIEKIAEH AVRKARAIWD LKEKLEVKLE ENEQYALYKE IELPLASILG TMESDGVLVD KQILVEMGHE LNIKLRAIEQ DIYALAGETF NINSPKQLGV ILFEKIGLTP IKKTKTGYST AADVLEKLAS EHEIIEQILL YRQLGKLNST YIEGLLKEIH EDDGKIHTRY QQALTSTGRL SSINPNLQNI PVRLEEGRKI RKAFVPSQPG WVMFAADYSQ IELRVLAHMS EDENLVEAFN NDLDIHTKTA MDVFHVEQEA VTSDMRRAAK AVNFGIVYGI SDYGLSQNLD ITRKEAATFI ENYLNSFPGV KGYMDDIVQD AKQTGYVTTI LNRRRYLPEI TSSNFNLRSF AERTAMNTPI QGSAADIIKK AMIDMAERLI SENMQTKMLL QVHDELIFEA PPEEIAMLEK IVPEVMENAI KLIVPLKVDY AFGSSWYDTK
